# Non-evacuation to cyclone shelters and the imperative of implementing disaster-resilient community-based housing and shelter plans

**DOI:** 10.4102/jamba.v18i1.2081

**Published:** 2026-09-25

**Authors:** Edris Alam

**Affiliations:** 1 Disaster Management Program, Faculty of Resilience, Rabdan Academy, Abu Dhabi, United Arab Emirates; 2 Disaster and Development Organization (DADO), Chittagong, Bangladesh

**Keywords:** cyclone emergency, safety, evacuation, sheltering options, disaster resilient housing, Bangladesh

## Abstract

Despite considerable progress in cyclone monitoring, early warning dissemination, evacuation planning and strengthening community confidence in alerts, one unresolved issue remains: why many residents opt not to evacuate during life-threatening events. A wide body of research has explored the factors influencing the hesitation of Bangladesh’s coastal communities to seek refuge in cyclone shelters during emergencies. Drawing on primary data obtained through in-depth interviews, focus group discussions (FGDs), participant observation and field visits, this study examines residents’ choices of shelter during recent cyclones in Bangladesh. In addition, it highlights a community-driven proposal for a sustainable solution. The study reveals a significant improvement in public trust towards cyclone warnings in Bangladesh, with confidence levels increasing from 56% during the 1991 cyclone to 96% by the time of Cyclone Amphan in 2020. The main reasons people choose not to evacuate during cyclones include the presence of stronger housing, worries about conditions in public shelters, the need to protect livelihoods and belongings, limited transport and logistical barriers, personal perceptions and attitudes, and issues related to the nature of cyclone warnings.

**Contribution:**

This study investigates the underlying reasons for residents’ reluctance to evacuate, focusing on their tendency to remain at home or move to nearby durable buildings. Such avoidance of cyclone shelters, even after warnings, underscores the urgency of developing community-based housing and shelter systems with disaster resilience. We recommend a housing model that addresses essential needs while aligning with the Sustainable Development Goals 2015–2030 (SDGs 3, 6, 7, and 13).

## Introduction

Globally, research on different hazards – such as floods, cyclones, landslides and volcanic eruptions – has examined why people ignore evacuation orders (Acero, Diaz & Behr 2023; Bakhshian & Martinez-Pastor 2023; Perera et al. 2020; Yore & Walker 2021). Key factors include distrust in warnings, low risk perception, limited awareness and education, transport disruption, scarcity of shelters, and inadequate space and privacy within them (Alam 2024). Despite Bangladesh’s success in decreasing the fatalities resulting from tropical cyclones (TCs) and the accompanying storm surges, certain coastal inhabitants are reluctant to adhere to cyclone evacuation directives. This reluctance constrains the effectiveness of disaster risk management efforts in the region (Alam 2024; Alam et al. 2024). Even with enhancements in TC detection and prediction, the timely provision of warning signals, the expansion of shelter centres, and arranging transport for evacuation along with food and essential assistance, a significant question arises: what causes certain coastal residents to disregard evacuation directives, and what do they prefer as shelter centres during cyclone emergencies? Previous studies have examined the evacuation patterns of residents in the aftermath of significant TCs dating back to the 1970s (Chakma & Hokugo 2020; Haque 1995; Parvin et al. 2019). These studies explored a range of problems associated with evacuation and provided remedial measures to overcome them, expecting that the community will follow evacuation orders if these problems are overcome. Although many of these issues have improved, shelter use during evacuations remains limited. Limited qualitative studies have examined intergenerational insights regarding residential preferences in cyclone-prone areas. In particular, there is a lack of data on community-led alternatives to distant relocation, including the feasibility of localised, disaster-resilient housing solutions. This study investigates evacuation behaviour and analyses community preferences for cyclone shelters along coastal Bangladesh; we focused on coastal inhabitants aged 59 and above who have experienced multiple TCs since the 1970s. Conceptually, this cohort was selected not merely as a ‘vulnerable group’ but as ‘knowledge holders’ whose lived experiences encompass multiple major cyclonic events (1970, 1985 and 1991).

### Cyclone early warning and public cyclone shelters

After the catastrophic 1970 Bhola cyclone, the Government of Bangladesh (GoB) initiated early warning systems (EWS) to protect people and assets from future cyclones. The Bangladesh Meteorological Department (BMD) has issued comprehensive bulletins that encompass the paths, strengths and predictions regarding where cyclones could make landfall since the 1980s. The BMD generates cyclone forecasts from radar, satellite and field data, serving as the main warning authority (BMD 2017). Communities are alerted through media, loudspeakers, flags, sirens, local networks and mosque announcements, ensuring broad coverage (Ahsan et al. 2020; Paul & Dutt 2010). Since 2009, mobile technology has further strengthened early warning dissemination in Bangladesh. The interactive voice response (IVR) services administered under the BMD enable the dissemination of early alerts sent to all callers. This platform also offers unrestricted and continuous access around the clock to essential information, including weather predictions on a daily basis, rainfall trends, TCs, flooding events and occurrences of landslides (Ministry of Disaster Management and Relief [MoDMR] 2018).

After the cyclones of 1960 and 1961 that caused around 20 000 deaths, the government built 132 two-storey facilities to function as union council offices, with the upper floors designated for cyclone shelter use. Following the 1970 Bhola cyclone, efforts to strengthen protection in coastal areas expanded through the construction of multipurpose shelters (see Figure 1). These typically feature open ground levels with the first floor raised about 3.7 m. Construction was at times halted or abandoned but was revived after each severe cyclone. Following the 1991 cyclone, by following suggestions from a technical committee, it was determined that governmental offices in regions susceptible to storm surges would be built as two-storey structures. This strategic design allowed the upper level to function as a refuge during emergency periods, enhancing overall resilience (Institute of the Engineers of Bangladesh [IEB] 1991).

**FIGURE 1:**
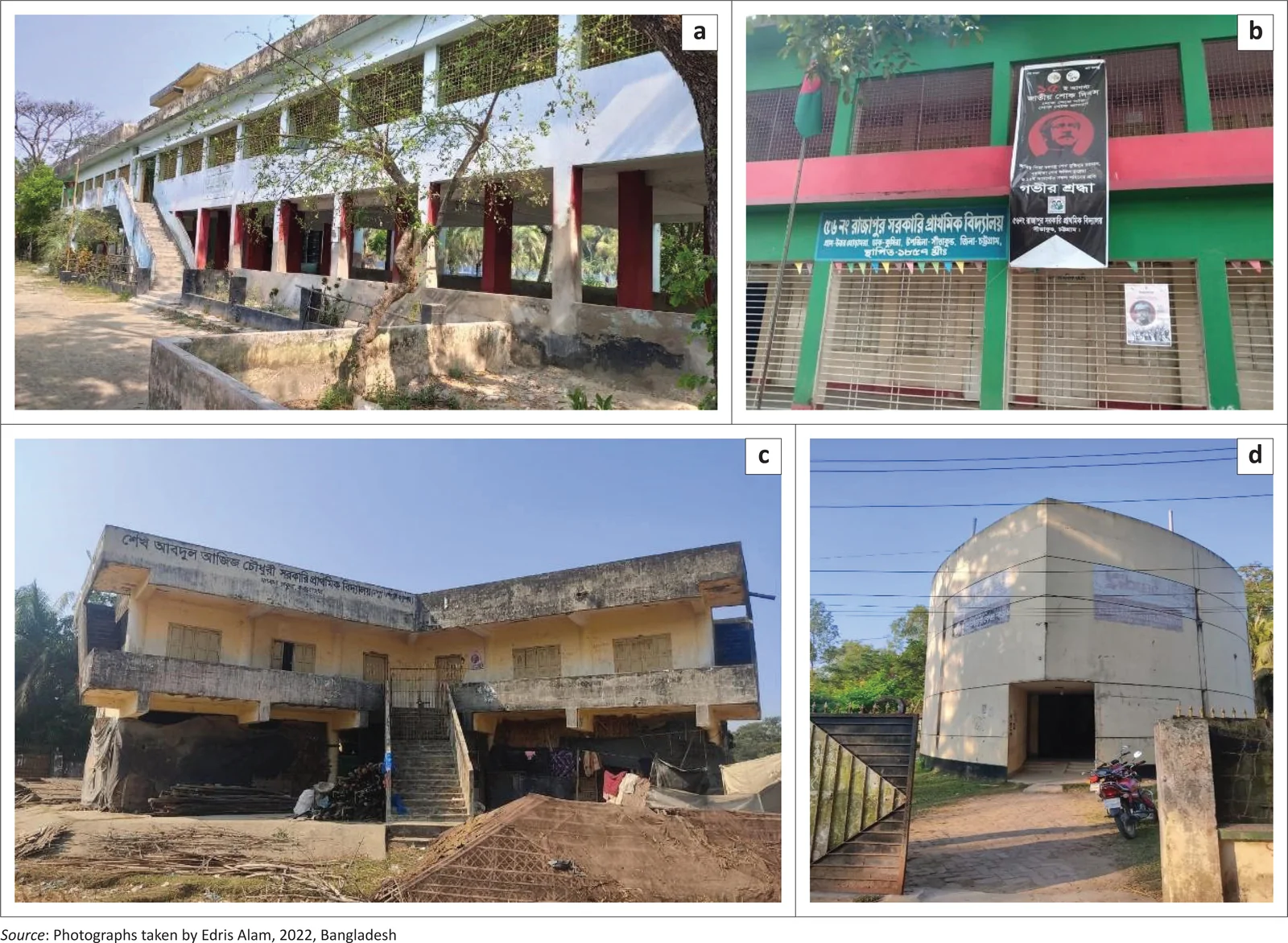
Photos depicting a variety of types of cyclone shelters along the Bangladesh coast: (a) public shelter centre in Khulna; (b) school serving as a shelter in Sitakunda; (c) public shelter centre in Pekua; and (d) Public cyclone shelter in Patuakhali.

To protect livestock, elevated structures called ‘killas’ were built higher than expected storm surges. Yet, isolated killas far from settlements were seldom used, as people were reluctant to travel long distances with animals, although each could hold about 350. Combined shelter–Killa designs, first introduced after Cyclone Gorky in 1991, became more effective (Swiss Disaster Relief 1993). At present, the MoDMR continues upgrading old killas and constructing new ones.

Despite advancements in shelter infrastructure, public centres are often underutilised during cyclone emergencies (Chakma & Hokugo 2020). This instigates the development of qualitative insights into the evacuation preferences of coastal communities in Bangladesh, bridging the gap between cyclone shelter provision and the actual preferences of resident communities.

### Framing evacuation behaviour and shelter management in disaster risk reduction mainstreaming

The existing literature extensively explores the public’s reaction to warnings and the perception of risk during emergency events. This body of literature encompasses studies addressing a range of hazards such as seismic events, TCs, nuclear energy and diseases. It incorporates both theoretical and practical viewpoints (Lindell & Perry 2012; Mileti & Peek 2000; Slovic 2000; Slovic et al. 2005). Numerous conceptual frameworks exploring disaster preparedness and responses carry considerable importance. Lindell and Perry (2012) proposed the Protective Action Decision Model (PADM) that outlines three crucial preliminary stages before making a decision: receiving it, paying attention to them and understanding it before further actions. By structuring the empirical analysis around these PADM components, the study identifies which stages of the decision process act as primary bottlenecks in the Bangladesh context – for example, whether non-evacuation is driven more by risk underestimation, distrust in warnings or dissatisfaction with shelter facilities. In parallel, the Crisis Cognition and Response framework by Comfort (2007) operationalised cognition as the capacity of local disaster management committees to interpret risk information, mobilise resources and coordinate evacuation logistics. Empirical indicators include inter-agency communication, leadership effectiveness and the integration of community feedback into response planning. This allows the study to link individual evacuation behaviour with systemic governance performance.

The broader Disaster Risk Reduction (DRR) framework is used to situate these behavioural and institutional dynamics within the disaster–development nexus. In this study, DRR mainstreaming is empirically examined through the availability, accessibility, and condition of cyclone shelters as indicators of infrastructure and service provision; livelihood dependence, poverty levels, and housing quality as dimensions of socio-economic vulnerability; and local coordination mechanisms, resource allocation, and policy implementation as measures of institutional capacity.

These variables are incorporated into the analytical model to explain spatial and social variation in evacuation outcomes across coastal communities. For instance, differences in evacuation rates are interpreted not only through individual decision-making (PADM) but also through structural constraints such as inadequate shelter capacity or weak institutional coordination, consistent with critiques of linear DRR approaches (Collins 2009; Twigg 2004).

Cyclone vulnerability in coastal Bangladesh is therefore conceptualised as an interaction between behavioural processes and development conditions. Empirical findings demonstrate that evacuation decisions are not solely a function of risk perception but are mediated by livelihood insecurity, social norms, gendered constraints and prior experiences with shelter environments. This supports the argument that disasters and development are mutually constitutive: while hazards disrupt development trajectories, underlying development deficits – such as inequitable access to safe infrastructure – shape disaster outcomes (Collins 2009, 2018).

By explicitly linking PADM to household-level behaviour, crisis cognition to institutional performance and DRR mainstreaming to structural conditions, the study advances a multiscalar analytical framework. This integration enables a more precise interpretation of evacuation behaviour in coastal Bangladesh and contributes to theoretical refinement by demonstrating how established models operate within resource-constrained, high-risk environments.

### Prior research on the evacuation patterns of coastal Bangladesh residents

While the history of disaster management in Bangladesh is extensive, a critical synthesis reveals a persistent gap between technological advancements in EWS and the behavioural realities of coastal residents. Islam (1974) conducted field visits and investigations, highlighting the community’s resilience and options for living with cyclone hazards. The research emphasised the imperative for constructing coastal embankments and conducting risk mapping. By examining cyclones from 1963, 1965 and 1970, Khan (1974) proposed comprehension of how risk perception influences community reactions to the upcoming cyclone hazards. The findings of this study revealed a deficiency in confidence in issued warnings among residents, inadequate preparedness and reliance on instinctual survival tactics to live through such events.

The devastating 1985 Urir Char TC, resulting in 11 000 casualties (Murty, Flather & Henry 1986), marked the country’s first major cyclone disaster that garnered national attention in post-independence Bangladesh. Despite early identification of a severe TC genesis on 23 May, 1985, and timely alerts issued towards the communities along the coast, local preparedness and response to the warning were inadequate. This led to a significant loss of lives on the islands of Sandwip and Urir Char, situated in the trajectory of TC landfall. Various factors contributed to the community’s non-response to the warning, including the considerable distance to public cyclone shelters, undervaluing the severity of the hazard, limited cyclone knowledge and divergent viewpoints on the necessity of evacuation (Siddique & Eusof 1987). In addition, community evacuation responses were influenced by structural factors such as residents’ age, sex and means of livelihood. For instance, resource-scarce families often prioritised the welfare of household animals and opted to stay home, believing they could protect them. Moreover, women typically deferred to male family heads, who traditionally made crucial decisions during emergencies (Alam & Collins 2010).

Following Bangladesh’s independence, the 1991 Cyclone Gorky emerged as the most devastating natural hazard, impacting over 138 000 individuals along the coastal regions (Alam et al. 2023). Despite successful dissemination of TC warnings, local communities failed to respond effectively because of distrust in the warnings, concerns about robbery and unavailability of public cyclone shelters (Haque & Blair 1992). Consequently, these factors contributed to a significant number of fatalities. The strength of TCs and the constrained ability of community members to respond, combined with an inefficient emergency response system, are pivotal elements amplifying the repercussions of cyclone devastation (Ohiduzzaman 1993). Moreover, reluctance among people to evacuate ahead of cyclone events stems from various reasons such as distrust in early warnings, inefficiencies in warning dissemination systems, a lack of accessibility to cyclone shelters and settlements, and inadequate administration of existing public shelter centres (Nur, Rahim & Rasheduzzaman 2021). To accommodate a larger number of coastal inhabitants, multipurpose buildings strategically located in high-risk areas were constructed. Despite this, the prevalent reluctance among residents to utilise these shelters during cyclones poses substantial challenges.

Over the last three decades, we witnessed notable advancements in warning dissemination, resulting in the successful evacuation of a larger portion of residents prior to cyclone landfall. These improvements likely contributed to the decrease in fatalities during TCs in coastal Bangladesh. Since Cyclone Gorky in 1991, only one category-4 cyclone, Cyclone Sidr, in 2007, made landfall on the western coast of Bangladesh, causing fewer than 5000 deaths. The relatively low death toll associated with Cyclone Sidr in 2007 was attributed to the measures to mitigate the risks associated with cyclone disasters in Bangladesh. Following Cyclone Sidr, Bangladesh did not experience any category-4 TCs making landfall. Between 2013 and 2017, the coastal region of Bangladesh experienced the impact of four Category-1 cyclones. The cyclones were identified as Mahasen (2013), Komen (2015), Roanu (2016) and Mora (2017). The combined effect of these cyclones resulted in casualties: 17 from Mahasen, 45 from Komen, 26 from Roanu and 6 from Mora. The factors contributing to the decision not to evacuate during these cyclones resembled those seen during the TCs that occurred in 1991 (Cyclone Gorky) and 2007 (Cyclone Sidr) (Ibrahim, Rahman & Rezwana 2019; Swarnali & Akihiko 2020). Existing literature has largely investigated these events in isolation, focusing on single-event post-mortems. There is a lack of longitudinal perspective that synthesises the lived experiences of survivors who have navigated multiple cycles of disaster since the 1970s. This study addresses this gap by utilising first-hand interviews to critically examine why, after five decades of institutional learning, the decision to refrain from seeking refuge remains a dominant response. In doing so, this study moves beyond merely identifying specific barriers to understanding how they have evolved – or persisted – within the collective consciousness of coastal communities. Furthermore, identifying disaster-resilient individual housing presents a sustainable pathway when integrated with projected livelihood opportunities, improved sanitation and solar energy solutions.

## Research methods and design

Qualitative techniques, comprising detailed interviews, discussions and observations, offer effective means for comprehending the populace’s reactions to natural hazards (Norris 2006; Phillips 1997). To gain insights into the challenges associated with evacuation, practical aspects during cyclone emergencies and the community’s preferences for cyclone sheltering, we conducted a total of 362 in-person interviews along with four focus group sessions involving community members between December 2021 and March 2022. The participants in these interviews and focus group discussions (FGDs) had first-hand experience with TCs in the years 1970, 1985, 1991, 2009, 2013, 2015, 2016, 2017 and 2020. The study methods employed in this research maintain the standards of social research norms while upholding the rights of the participants.

For field visits, investigations, and surveys, 15 study areas were chosen along the Bangladesh coast (see Figure 2). These areas are recognised as some of the most frequently affected cyclone-prone regions in Bangladesh (Alam et al. 2024). Prior to conducting the interviews, we compiled a list of potential participants, focusing on the acceptance of the invitation and availability of the household heads as the primary criteria for selection. Following this inventory, 362 in-person interviews were randomly carried out with household heads who were above 59 years of age, who had encountered severe TCs, and who resided within a 3 km distance of the coast. These specific criteria were chosen because household heads within this demographic were deemed to have heightened knowledge of living with TCs. It was found that individuals residing within a 3 km radius of the coast were most significantly impacted by previous cyclonic occurrences (Alam 2003, 2023).

**FIGURE 2:**
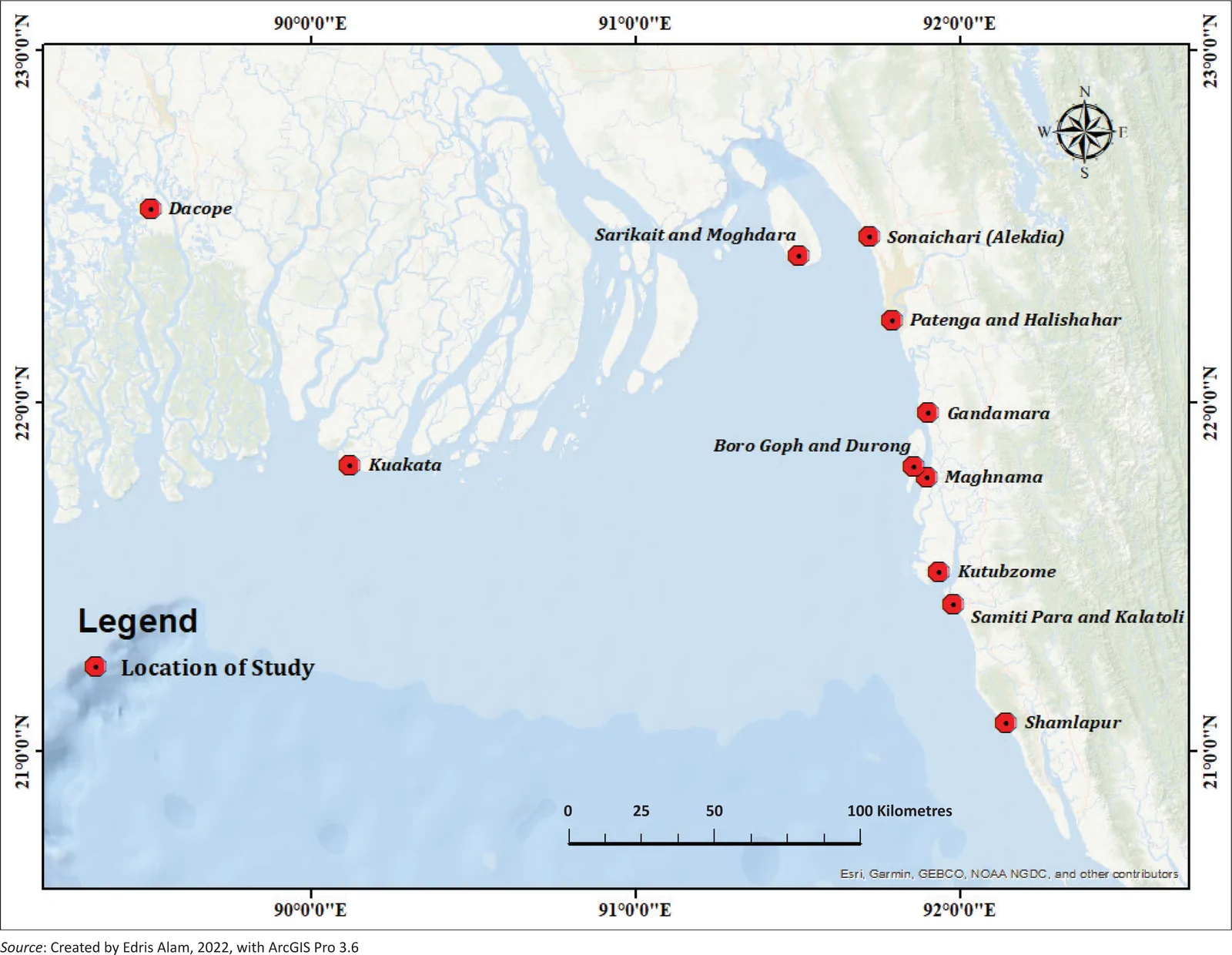
Map depicting the locations of field visits, interviews and observations on the coastal Bangladesh.

The participants in this study had witnessed cyclones occurring between 1960 and 2021. Respondents aged over 59 were specifically chosen for interviews, as they were deemed better equipped to provide insights into non-evacuation behaviour and community responses and share their experiences with cyclones. The interview questions are divided into three sections. The initial section consisted of structural enquiries regarding residents’ socio-demographic attributes, housing types and foundation designs, income, and educational attainment. The subsequent section focused on community responses to warnings, changes in emergency sheltering locations, reasons for non-compliance with warnings, and preferences for emergency sheltering. The final part included questions about the possibility and preference of community-based disaster-resilient housing on the Bangladesh coast.

Apart from conducting 362 in-person interviews, four FGDs were conducted. Participants in these discussions were coastal and island inhabitants aged over 59 who particularly varied aspects, including design, location and facilities to be included in community disaster-resilient housing. The schedule and venue for the FGDs were determined collaboratively with participants in advance. At the beginning of every FGD, following personal introductions, the organiser outlined the study objectives and invited the participants to provide their insights on different aspects of formal emergency sheltering and details on proposed community-based disaster-resilient housing. Subsequently, the organiser introduced participants’ rights and obtained consent from them. Of the four FGDs, three involved male participants engaged in professions such as farmers, fishers, day workers, or small traders or were retirees. The remaining FGD included women who were housewives. Every FGD comprised seven participants, and it took from 50 to 60 min for each meeting. The fundamental accounts emerging from these discussions offered clear insights into changes in the community’s attitude, offering insights on residents’ hazard responses, shelter-seeking behaviour and the imperative to develop disaster-resilient housing in Bangladesh.

At first, the field survey data underwent meticulous examination and was subsequently assembled for analysis. Subsequently, the collected data were analysed, with responses to open and non-structural questions categorised based on thematic evidence. In addition, averages were computed from participant responses. Open-ended interview responses were systematically and manually processed and analysed, applying Microsoft Word and Excel. The results were presented as a frequency tally, organised hierarchically based on their respective weight values. To identify emerging themes from the FGDs, we utilised NVivo software for content analysis, presenting the findings in a structured manner (Krippendorff 2018). The approach proposed in this study contributes to fostering a deeper understanding of the answers that correspond with the enquiries of the study. Moreover, the study employed an inductive coding process to systematically categorise themes reflecting comparable accounts, such as ‘I place confidence in alerts’, ‘I opt for remaining at home during a cyclone crisis’, ‘I favor seeking refuge in a sturdy nearby residence’, ‘Space concerns arise in cyclone shelters’ and ‘We seek assistance to build cyclone-resistant homes’. These codes were then elaborated into broader themes that corresponded with the study goals. The analysis of these results was informed by pertinent theoretical and developmental literature.

It is to be observed that the study relied on a convenience sampling approach, and an exclusive focus on household heads aged 59 and above may introduce selection bias. Our focus on the elderly cohort – while intentional to capture a longitudinal perspective of cyclone events since the 1970s – limits the generalisability of our findings to the broader coastal population. Specifically, the risk perceptions and evacuation behaviours of younger generations, who may possess higher digital literacy and different socio-economic priorities, remain outside the scope of this analysis. Consequently, while these findings offer deep historical and experiential insights, they should be interpreted as representative of a specific demographics’ lived reality rather than a universal trend across all coastal residents.

## Results

### Social and economic attributes of the participants

Out of the 362 participants, 293 were male (81%) and 69 were female (19%). This distribution highlights the infrequent occurrence of women as heads of households in the study area. The mean age of those who participated in the interviews was 66.87 years, as we exclusively surveyed individuals aged 59 years and above. The average number of family members was 6.47. Forty-seven per cent of respondents belong to nuclear families and the remaining 53% are from extended families. Regarding monthly income, the respondents reported an average of Bangladeshi Taka (BDT) 13 083. (*Note: On 19 April, 2023, the exchange rate was 1 United States Dollar [USD] equals 109.70 BDT). Overall, those who live close to the coast are a lower-income group. The income distribution of 362 households in seven income ranges, starting from BDT 0–5000, 5001–10 000, 10 001–15 000, 15 001–20 000, 20 001–25 000, 25 001–30 000, and above 30 000, were 16%, 26%, 33%, 17%, 5%, 4%, and 6%, respectively. Despite Bangladesh having a literacy rate of 76% in 2022, approximately 51% of our survey respondents were unable to read or write, as this study exclusively targeted individuals aged 59 years and older. Among the remaining 49% of participants, educational attainment varied: 40.61% had completed primary education (1–5 years of schooling), 1.38% had finished lower secondary education (6–8 years of schooling), 2.76% had attained a secondary school certificate (SSC), 2.76% had obtained a higher secondary school certificate (HSC), while 1.11% had graduated from college or university. Approximately one-third of the participants are involved in agricultural activities, and fishing and day labour represent 14% and 13%, respectively. The remaining respondents were involved in small-scale trading, homemaking, fish trading, service provision, expatriate work and business. While none of the participants primarily relied on livestock rearing as their occupation, 33% of them were involved in it as a supplementary source of income.

### Housing patterns

Amid intense cyclonic occurrences and ensuing storm surges, the presence of sturdy houses stands out as a crucial factor contributing to human survival. Earlier, only 3.32% of participants resided in single-storey structures made of semibrick or brick, whereas presently, this figure has risen to 17.13%, indicating enhancement in housing structures. This also meant that currently roughly 82.87% reside in various types of kutcha (non-built) houses: those made of tin, tin and wood, straw and polyethylene. Overall, there has been a reduction in the quantity of kutcha houses (Figure 3). In Bangladesh, prior to the introduction of newer housing types, around 41% of inhabitants lived in residences characterised by frail constructions, often made of straw and bamboo, featuring roofs crafted from paddy straw. Research shows that merely 11% of respondents presently inhabit such dwellings. The foundation (ground-level base and beam) of a house plays a crucial role in enhancing its resilience and reinforcing its ability to endure storm surges. Currently, there are still 81% plinths of houses made of mud, whereas it was 93% in the past. Currently, 12% of plinths are brick-built, compared with only 3% before Cyclone Gorky in 1991. Overall, there has been a decrease in houses solely constructed from mud (earthen), accompanied by an increase in those featuring brick-built and semi-brick-built plinths. The rise of brick-built houses is slow because the average income of households is BDT 13 083 and only 6% of households earned above BDT 30 000.

**FIGURE 3:**
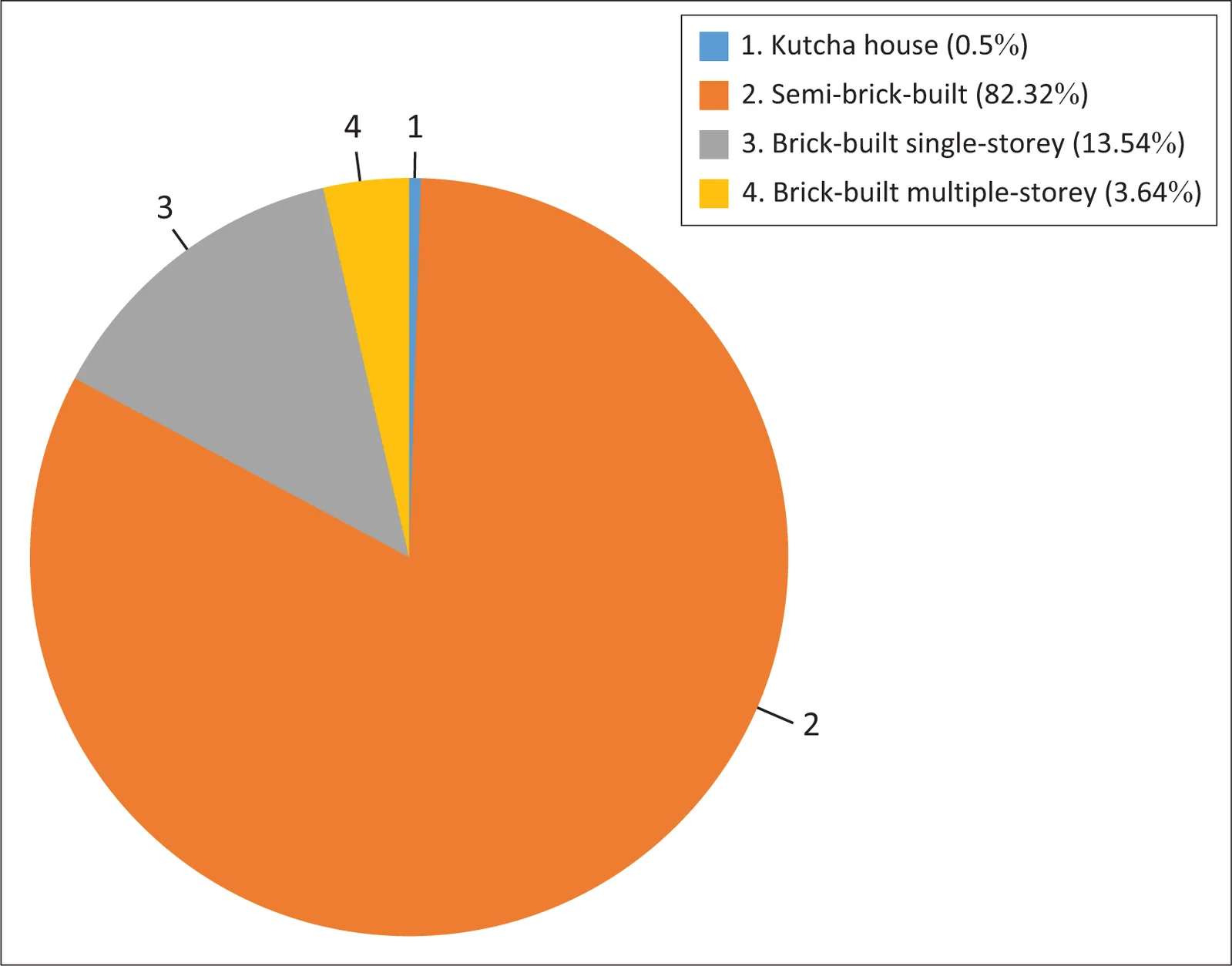
The number of participants living in four different types of houses, including (1) kutcha, (2) semi-brick-built, (3) brick-built single-storey and (4) brick-built multiple-storey houses in the study areas.

### Responses to cyclone alerts and evacuation orders

The research suggests a significant rise in the households’ reliance on cyclone emergency alerts. Out of the 362 individuals, 94% displayed confidence in emergency warnings, while the rest, 6%, lacked enough trust to evacuate the vicinity (Figure 4). During Cyclone Gorky in 1991, 54% of residents trusted the warnings, with 46% harboured scepticism. Generally, individuals lacking sturdy housing preferred moving to cyclone shelters. Out of the participants, 63% carried out an evacuation during the most recent severe cyclone, seeking refuge in various resilient locations such as public cyclone centres, neighbouring robust houses, religious institutions, medical facilities and other public or private structures. Surveys regarding housing revealed that 18% of participants resided in sturdy homes and therefore opted not to evacuate during cyclone emergencies. Notably, 37% of residents opted not to evacuate, with the specific reasons detailed in Discussion and Conclusion sections. Residents who had experienced family losses during cyclones were more inclined to heed evacuation orders, especially upon receiving warnings of signals ≥ 6. Consequently, these individuals demonstrated a higher propensity to evacuate compared to those who had not experienced casualties.

**FIGURE 4:**
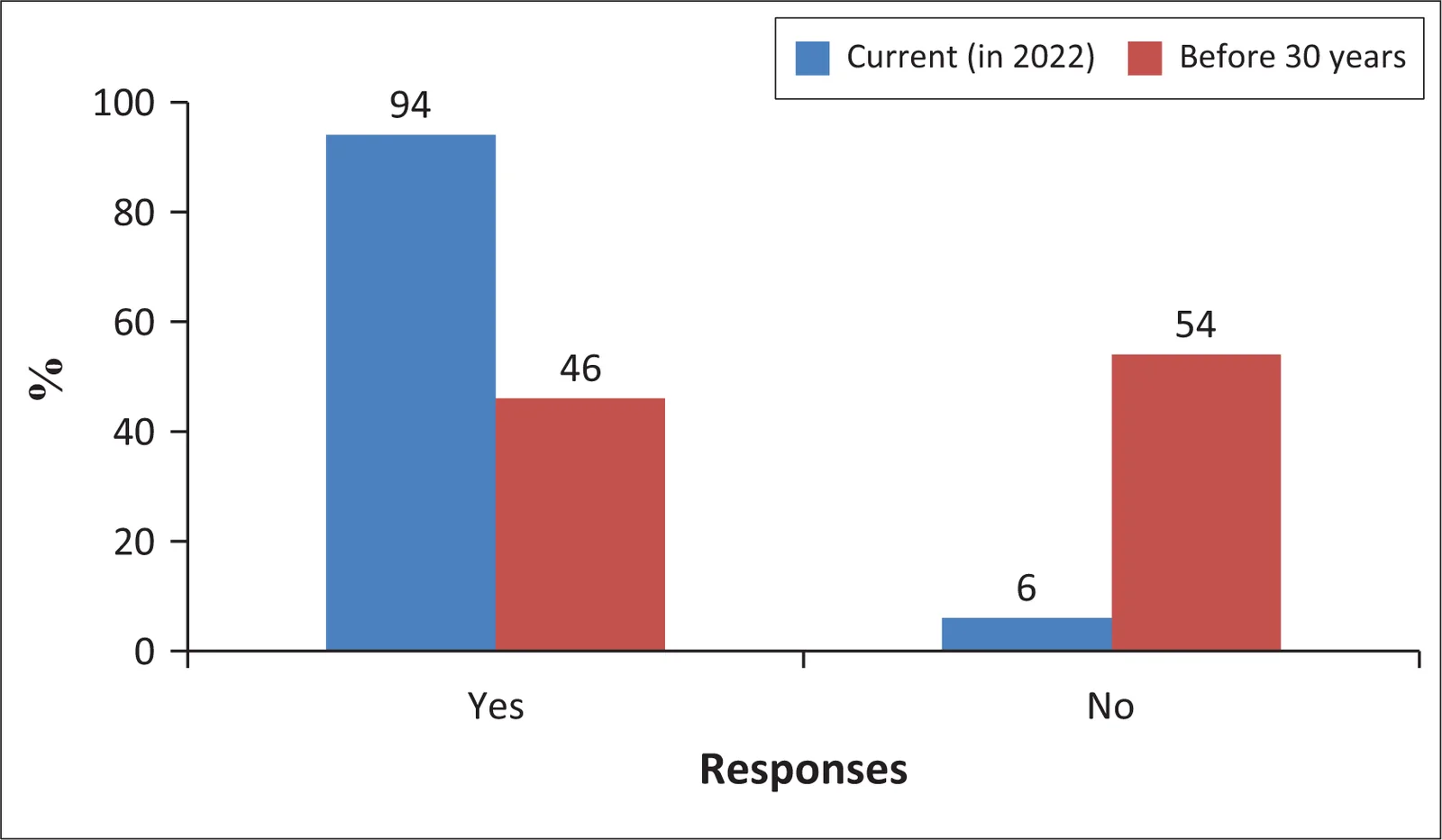
Trust in warning in 2022 versus about 30 years ago (in 1991).

### Non-compliance with cyclone warning and past behaviour of seeking refuge and shelter during severe cyclones

The findings suggest that individuals residing in kutcha (non-built-in) houses move the members of their household to reside with extended family members who have more durable houses. Generally, numerous inhabitants sought shelter with their in-laws living further inland, away from the coastline. Significantly, there were considerable differences in the behaviour of seeking shelter among participants, which were influenced by the geographical location of their residences, prior encounters with TCs and the frequency of such occurrences. There has been a broadening of the options for sheltering, encompassing school buildings, hospitals, mosques and nearby residences. Participants from Sandwip and Sitakunda explained that, during the 2017 Cyclone Mora, they preferred staying in their neighbours’ homes. The participants noticed a rise in the quantity of strengthened buildings in proximity to their residences. Given the promptness in the intensification of cyclone severity, they could promptly seek shelter in these reinforced infrastructures, bypassing the need to travel to public shelters and congregate with others. In regions lacking public shelter centres or where these centres were at a considerable distance, these structures were the only available choice for seeking refuge. In a few instances, participants highlighted the absence of formal shelter in their vicinity but mentioned the presence of well-constructed mosques, temples and health centres that were utilised for refuge during the cyclonic season. A resident in Cox’s Bazar stated: ‘I can find shelter in nearby residences, mosques, or other sturdy structures if there’s a storm surge’. It is worth noting that just 3.32% of residents resided in single-storey houses constructed of semi-brick or brick before; the study revealed that 17.13% currently resided in similar housing categories. A participant in Sitakunda opined: ‘I have a durable and robust house’.

In regard to the open-ended query regarding reasons for not evacuating during the recent severe cyclone alert, we collected a sum of 898 answers. These responses were then organised into 10 primary reasons: (1) accessibility to nearby masonry structures; (2) limited space; (3) attachment to pets and safety; (4) challenges relocating family members; (5) having of established homes; (6) limited vehicles for evacuation; (7) distance to designated shelters; (8) concerns about household theft; (9) absence of recent encounters with significant TCs; (10) unwavering trust in Allah (God). We have found several direct quotes as lived experiences of participants as major outcomes of community consultation regarding non-evacuation to cyclone shelters following early warning dissemination. A resident in Sandwip uttered from the past experience, ‘Issues within public shelter centres include space constraints, limited personal space, and inadequate sanitation facilities’.

Some residents voiced concerns regarding space limitations within shelter centres and inadequate bathroom amenities. Residents of Sandwip Island and Maheskhali expressed worries specifically regarding the privacy of women in communal shelter centres. Instances of harassment, described as ‘jorajari’ [*entanglement*] and ‘gathagadi’ [*aggregation*], were either experienced first-hand or heard of by participants. They found that individuals living close to shelter centres tended to occupy available space, causing difficulties for others. In Maheskhali, 30.30% of participants (10 out of 33 respondents) stated that they had to go back home because the public shelter centres lacked adequate space. A participant from Sarikait in Sandwip: ‘I was unable to find a spot in the public shelter during my previous attempt to enter’. Residents of Patenga observed that economically disadvantaged individuals moved to shelter centres because of inadequate housing and the belief that shelters might offer food and other assistance. In Kalatoli, participants mentioned the absence of nearby public shelters. Similarly, Kuakata residents noted the lack of public shelter centres in their area, with cyclones causing road inundation, hindering movement. A participant stated: ‘The public cyclone shelter is either too far away or there isn’t one in the area’. Fear of potential hazards such as falling trees, strong winds and rain during travel to public cyclone shelters led to a preference for staying at home, referred to locally as ‘bithe mati’ (the foundation of one’s own home). Despite evolving perceptions regarding sheltering, the inclination to prefer home shelters during emergencies still hinges on the proximity of public shelter centres to residences and road conditions. Evacuation following a TC warning signal presents challenges for agricultural communities, as they are reluctant to leave for cyclone shelters and have concerns about leaving their livestock behind. A resident who depends on rearing livestock for a living: ‘I cannot evacuate alone, especially if it means leaving behind domestic animals’.

Taking into account the practical aspects, participants underscored that seeking shelter was not feasible because of the various challenges linked with public cyclone shelters, as detailed earlier in this section. Discussions with eight participants, ages ranging from 45-55 years and mostly farmers in the region at Alekdia suggest that:

‘We experience storm surges multiple times in a season. At times, pregnant women face difficulties in evacuating when a disaster occurs. It is preferable to remain at home, even if it entails unfavourable outcomes.’

Participants from Kuakata and Alekdia highlighted that when relocating to a shelter centre, ‘there is a risk of theft of animals and belongings’.

Residents who live adjacent to coastal levees elaborated that amid cyclonic depressions, roads become flooded, shelters are often distant and transportation is unavailable. Unpaved dirt roads are commonly affected by rainwater, becoming slippery. In such challenging conditions, moving to a designated shelter with elderly people, women, and children becomes extremely difficult. Participants from Dacope and Sandwip highlighted these difficulties. ‘Trees topple onto the streets, obstructing roads. Individuals are apprehensive about sustaining injuries from falling trees and choose to seek safety in their homes’. On several occasions, participants noted a rise in the elderly population at home. In Sandwip, one participant cited the case of an individual with disability with restricted mobility, underscoring the considerable hurdles in relocating to a public shelter centre amid severe winds and rain, particularly with elderly and disabled individuals.

On multiple occasions, the intensity of cyclones was either overstated or understated. Residents from Sandwip and Maheskhali found that they often fail to receive warnings with adequate lead time. The findings from the FGDs revealed a common refrain: ‘Even though they warned of storm surge coming, it did not happen’. Instances of such a nature were frequent, leaving residents uncertain about the accuracy of the warning signals.

### A proposal for disaster-resilient community-based housing

Although cyclone winds and storm surges cause significant damage to housing in the region, residents do not evacuate to cyclone shelters despite substantial improvements in early warnings, evacuation logistics, and the number of formal cyclone shelters. To make a feasible solution to this long-standing problem, the community asks what they would prefer for safe refuge during a cyclone emergency in the future. Summation of responses from open-ended questions and FGDs can be categorised into two options: (1) disaster-resilient individual housing and (2) disaster-resilient community settlements. The first option is the gradual development of disaster-resilient housing instead of kutcha houses in the high storm surge areas. Considering the fact that a household living in a high-risk zone lost their houses several times over their lifetime, an investment in a disaster-resilient house is suggested to reduce deaths, injuries and poverty in the region. A typical disaster-resilient house is for a poor family, averaging seven persons in normal periods and can accommodate 40 people during cyclone emergency periods (Figure 5).

**FIGURE 5:**
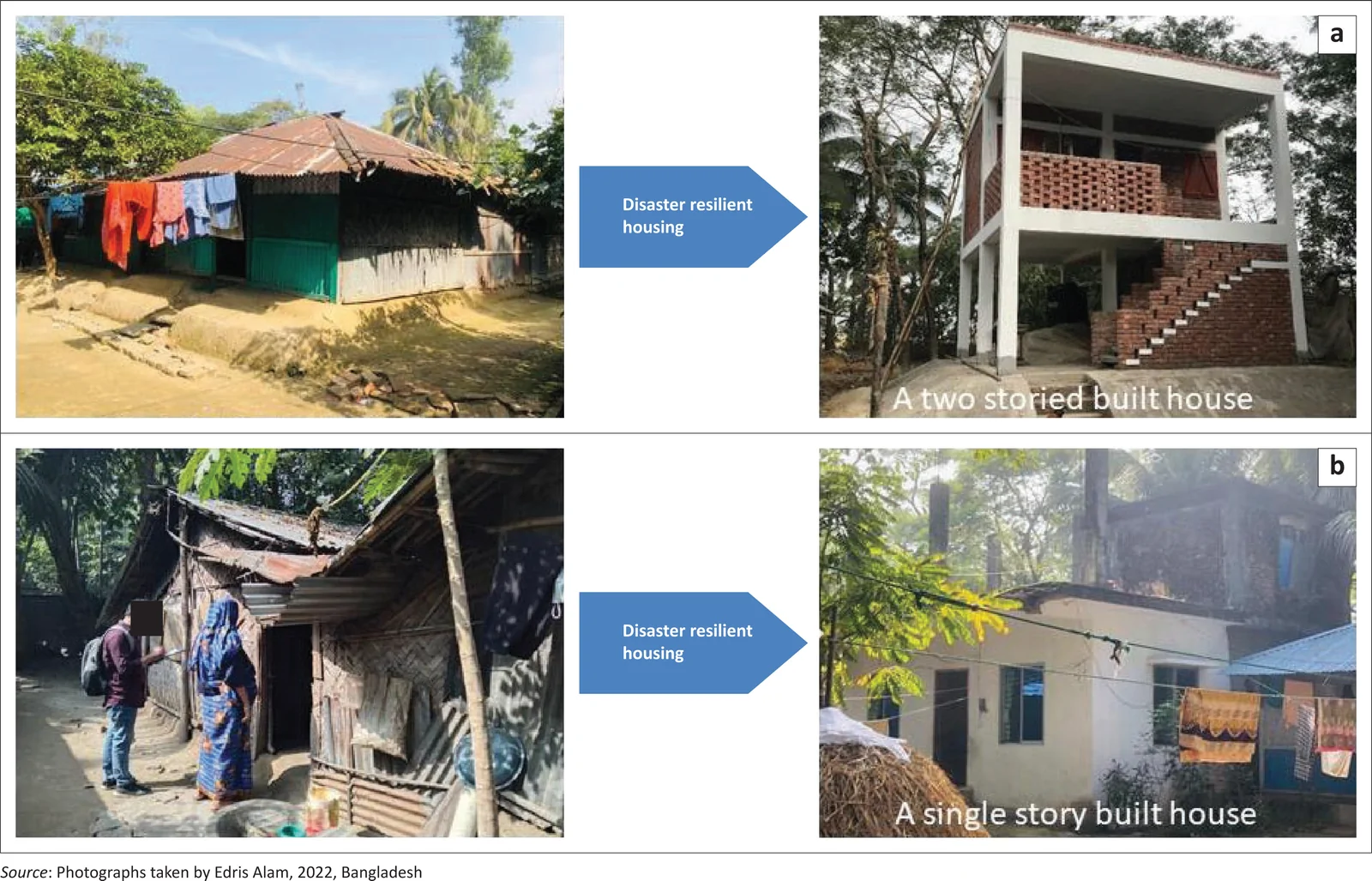
Disaster-resilient individual housing to reduce deaths and poverty and to enhance development: (a) a typical structure where the ground floor would be empty and the second floor would be used for living purposes. The ground place could also be used for livestock rearing; and (b) a second type of disaster-resilient housing where the ground floor will be used for living and an additional room on the first floor will be used for sheltering that family during an emergency period.

The community suggested developing a disaster-resilient community settlement, considering the expansion of a new settlement and also planning to develop a settlement in new emerging areas. The several key features of disaster-resilient community settlement include (Figure 6): (1) re-organise isolated people in newly constructed disaster-resilient sustainable settlement; (2) disaster-resilient houses; (3) access to growth centre, marketplace and livelihood opportunity; (4) appropriate training on disaster preparedness and recovery; (5) improved education and sanitation programme; (6) provision of solar panels instead of traditional biofuel and grid electricity; (7) access to safe drinking and bathing water and sanitation facilities; (8) protection of children and women; (9) poverty reduction through sustainable livelihood option; (10) housing for eight families and shelter for 320 people in emergency.

**FIGURE 6:**
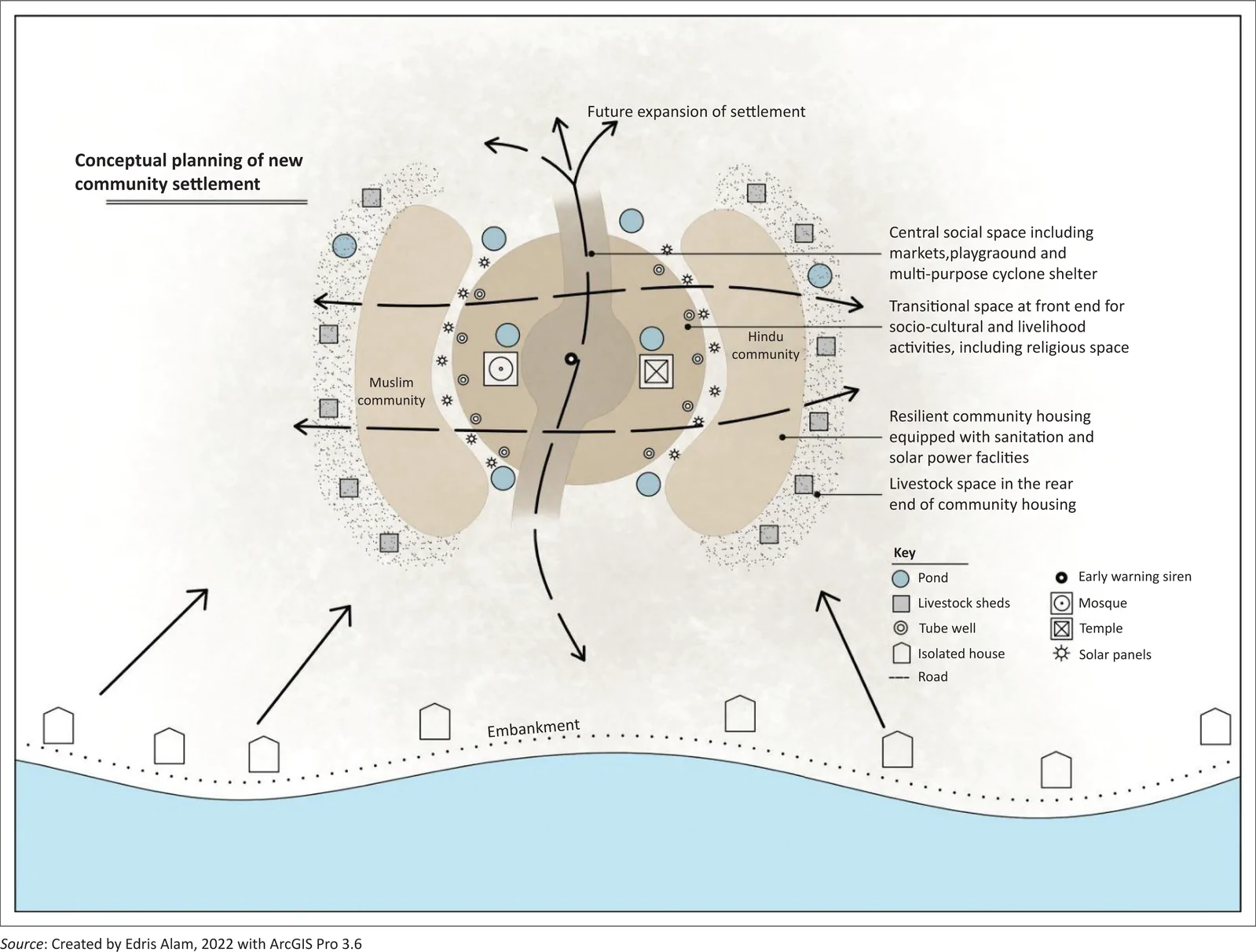
Disaster resilient community settlement allowing isolated houses into compact houses providing solar energy, sanitation, space for domestic animals and easy access to emergency shelter centre and market places.

## Discussion

Multiple donors, non-governmental organisations and entities are involved in funding the building and enhancement of cyclone shelters in Bangladesh. Through these collective efforts, there has been a notable surge in the quantity of cyclone shelters over the years, increasing from 44 in 1970 to 2500 in 2018 (Faruk, Ashraf & Ferdaus 2018). Despite these endeavours, the current shelters are inadequate to house the 35 million coastal inhabitants in coastal Bangladesh. The inadequacy and distance to designated shelters have been major factors contributing to the high casualty rates during cyclones (Haque 1995). Despite the presence of cyclone shelters, the rate of evacuation in response to emergency warnings and the number of coastal residents seeking shelter in these facilities remain low. For instance, the rates of failure to evacuate after the cyclones of 1970 and 1991 were 99% and 70%, respectively (Chowdhury et al. 1993; Frank & Husain 1971). Research conducted by Paul (2014) focused on examining evacuation behaviour during the cyclone Sidr of 2007. A study by Ahsan et al. (2016) investigated evacuation patterns during the cyclone Aila in 2009. In addition, research by Saha and Pittock (2021) examined evacuation behaviour during the cyclone Mora in 2017. These investigations revealed rates of failure to evacuate at 57%, 74% and 78%, respectively (Table 1). The proportion of evacuees during the cyclone emergency remained notably minimal, not exceeding 20% (Parvin et al. 2019). A review outcome of the evacuation rate of residents during cyclone emergencies in Bangladesh has been presented in Table 1.

**TABLE 1:** The evacuation actions of residents during cyclone emergency in Bangladesh.

Evacuation behaviours Year	Intensity of tropical cyclone in SSHWS**	Non-evacuation	Evacuation
1970 Cyclone Bhola (Frank & Husain 1971)	4	> 99	< 1
1991 Cyclone Gorky (Chowdhury et al. 1993)	4	1390 (69.5)	610 (30.5)
2007 Cyclone Sidr (Paul 2014)	4	194 (58.6)	137 (41.4)
2009 Cyclone Aila (Ahsan et al. 2016)	1	311 (74)	109 (26)
2017 Cyclone Mora (Saha & Pittock 2021)	1	39 (39)	61 (61)
Alam (2024)	-	134 (37)	228 (63)

Note: Please see the full reference list of this article: Alam, E., 2026, ‘Non-evacuation to cyclone shelters and the imperative of implementing disaster-resilient community-based housing and shelter plans’, *Jàmbá: Journal of Disaster Risk Studies* 18(1), a2081. https://doi.org/10.4102/jamba.v18i1.2081, for more information.

SSHWS, Saffir-Simpson hurricane wind scale.

Notes:

** , Saffir-Simpson hurricane wind scale (SSHWS). Numbers in parentheses denote the percentage distribution.

The findings of this study reveal a significant paradox in Bangladesh’s disaster management landscape: while the mean distance to formal cyclone shelters has decreased from 4 km to approximately 1 km over three decades, evacuation compliance remains suboptimal. This suggests that spatial proximity is no longer the primary determinant of shelter utilisation. Instead, our data indicate a transition towards informal and decentralised sheltering strategies, a phenomenon that both extends and complicates established theories of disaster response in the Global South.

While prior research has long emphasised the deficit of formal infrastructure (Paul & Dutt 2010), our findings demonstrate that residents in regions such as Chittagong and Cox’s Bazar have adapted through the utilisation of mosques, clinics and commercial high-rises (Figure 7). In tourism-heavy zones such as Kolatoli, the emergence of hotels as de facto shelters represents a shift towards private-sector-reliant resilience. This extends the ‘social capital’ framework of evacuation, suggesting that residents prioritise the structural integrity of nearby familiar buildings over the perceived safety of distant, formal shelters. This aligns with recent observations in Southeast Asia where ‘place-based’ resilience often supersedes state-mandated protocols (Alam et al. 2024).

**FIGURE 7:**
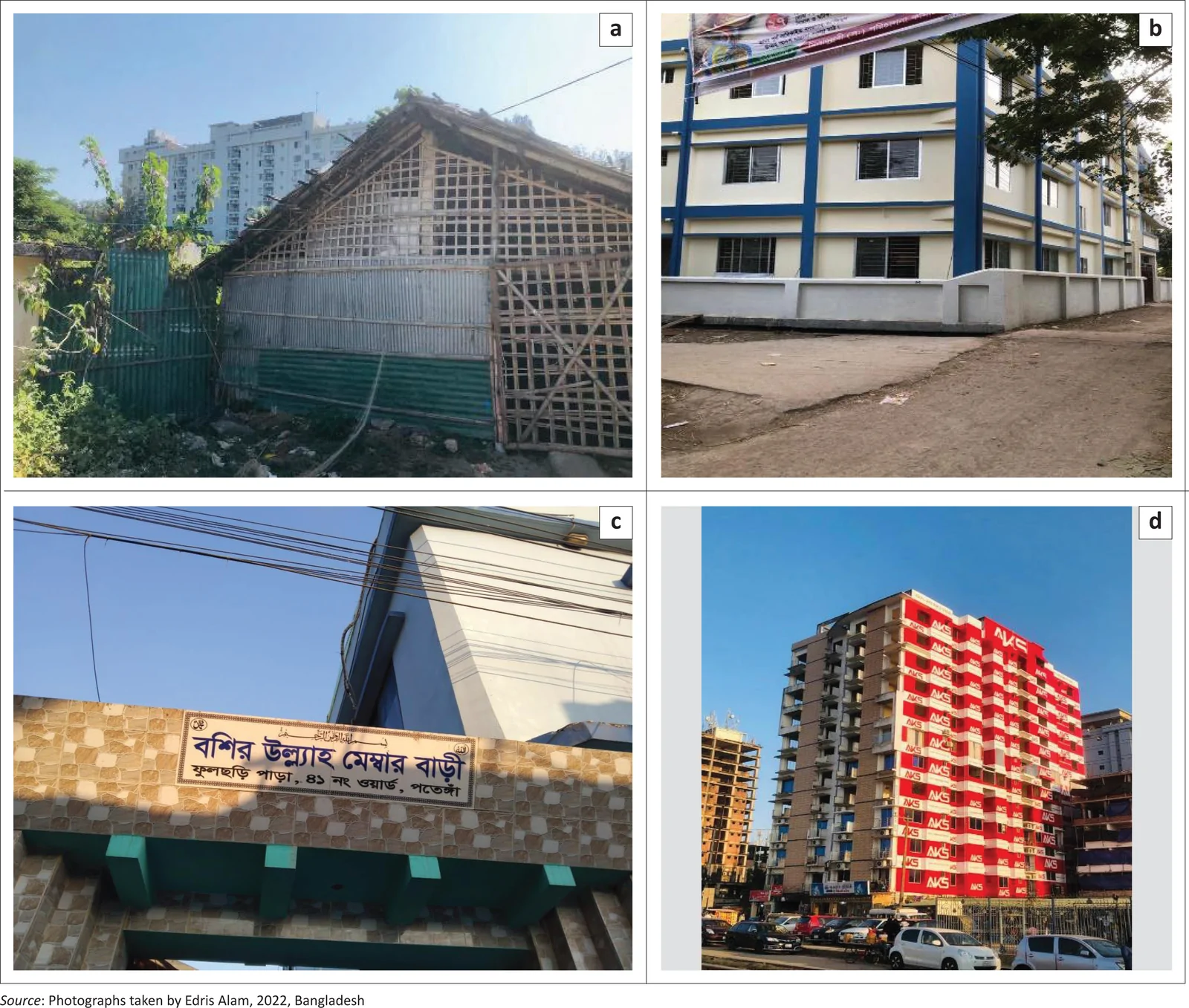
The presence of informal shelters within the study region: (a) low-income households residing near multi-storeyed buildings anticipate taking shelter in these nearby buildings; (b) private school building in Sandwip; (c) recently erected residential structures often used as a shelter in South Pantenga; and (d) multistoreyed commercial buildings in Cox’s Bazar.

Our results corroborate the findings of Alam (2024) regarding the prohibitive ‘hidden costs’ of evacuation. Beyond transportation, the recurring burden of lost wages and food insecurity during displacement creates an economic barrier that formal disaster policy frequently overlooks. The reluctance of household heads to evacuate – driven by the need to protect livestock and assets – reinforces the argument that for the coastal poor, the risk of post-disaster impoverishment is often perceived as greater than the immediate physical risk of the cyclone. This contradicts the traditional ‘rational actor’ model of evacuation, which assumes physical survival is the sole motivator (Lindell & Perry 2012).

The study identifies a critical ‘late-stage evacuation’ risk. While escalating warning signals eventually drive relocation, the physical environment often becomes impassable before the transition occurs. The documentation of injuries from uprooted trees and the destruction of unpaved roads during Cyclone Sidr (2007) highlights a persistent failure in last-mile connectivity. Unlike existing literature that focuses primarily on shelter capacity, our findings emphasise that the degradation of transportation corridors during landfalls renders even short distances (e.g. 500 m) insurmountable.

By synthesising field observations with socio-economic data, this study contributes a new dimension to the literature: We argue that vulnerability is not merely a product of poor housing but a systemic failure to align disaster infrastructure with the livelihood realities of the coastal population. Future risk reduction must pivot from constructing isolated shelters to developing resilient community clusters – where housing, livelihood protection (e.g. livestock pits) and transport infrastructure are jointly engineered to facilitate rapid, low-cost safety.

Household heads often opt against moving to cyclone shelters because of their attachment to pets, anxieties about property and the apprehension of losing their source of income. However, if the cyclone’s intensity escalates and the warning signals become more severe, families may reconsider and decide to evacuate their homes. Yet, at that point, it may be practically challenging to relocate because of the escalating wind speed and rainfall. Moreover, uprooted trees blocking access roads to cyclone shelters further hinder evacuation efforts. Furthermore, the joint impact of rainfall and wind can result in harm to or complete destruction of unpaved roads. Various injuries, ranging from cuts and bruises to fractures, caused by falling trees were documented during Cyclone Sidr in 2007. The apprehension of sustaining injuries from airborne objects, such as trees torn from their roots, vehicles, rubble and precarious building frameworks, also deters people from seeking refuge in cyclone shelters. In combination with interviews, FGDs, field visits and investigations, we identify several practical challenges, including poor road conditions, limited livelihood options and vulnerable dwellings, prevalent on the Bangladesh coast (Figure 8).

**FIGURE 8:**
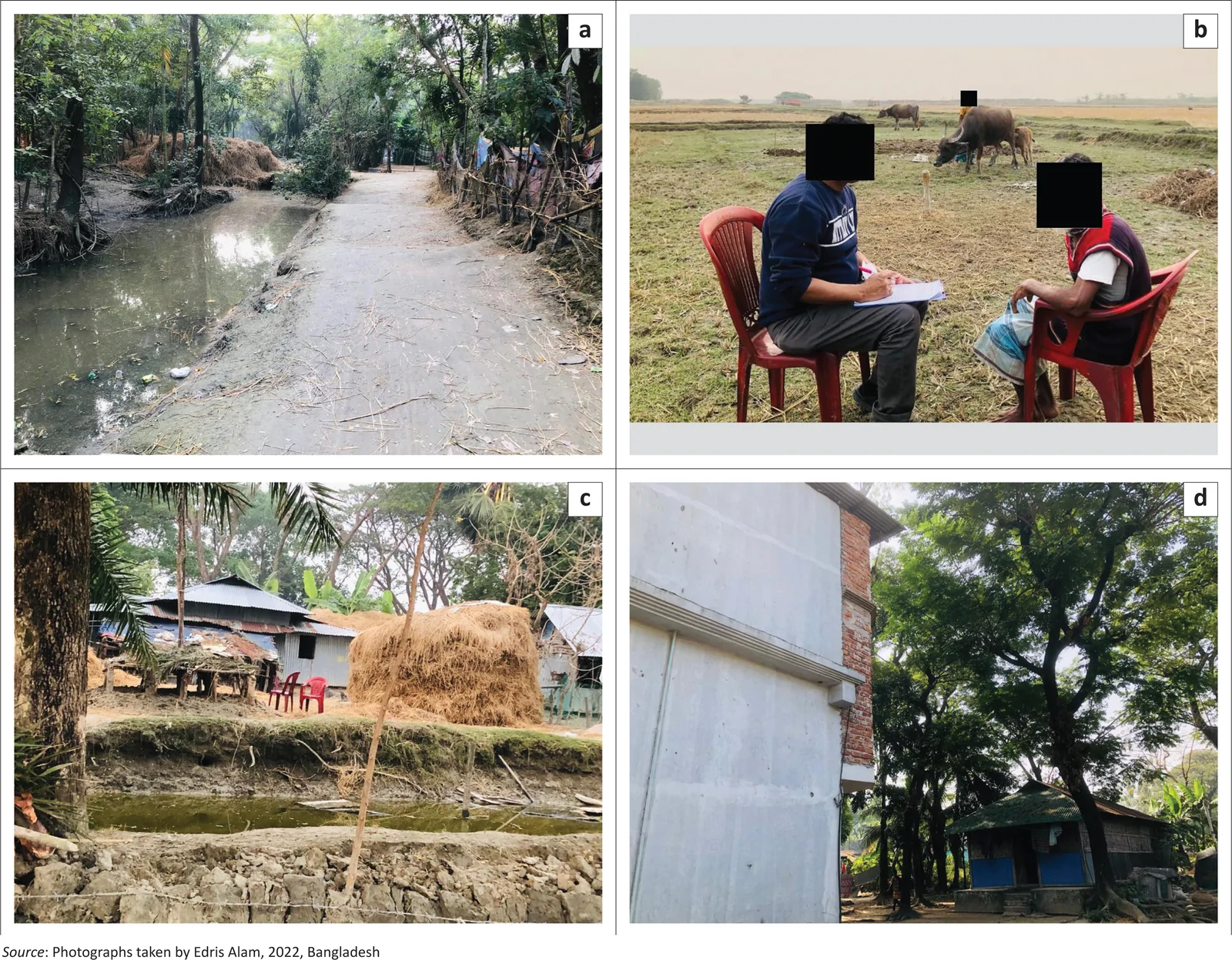
Practical challenges in the study sites along the Bangladesh coast: (a) Kutcha Roads: illustrates dilapidated and submerged roads in Sandwip, highlighting the difficulties of transportation during cyclones; (b) Livelihoods: depicts a respondent with domestic animals in Moghhara, symbolising the importance of domestic animals for livelihoods in coastal communities; (c) Housing and belongings: shows a residence along with its possessions, including provisions for livestock, underscoring the attachment of residents to their homes and possessions; and (d) Contrasting homes: presents an instance of a sturdy dwelling situated next to a delicate residence, there is scope for easy movement of residents from the latter to the former during an emergency period.

## Conclusion

Existing literature on cyclone alerts and evacuation in coastal Bangladesh has extensively addressed shelter accessibility, warning dissemination and demographic determinants (Alam et al. 2023; Paul & Dutt 2010). However, despite the proliferation of public shelters and reduced travel distances, evacuation rates remain stagnantly low. This study moves beyond traditional barriers to examine the socio-economic drivers of non-evacuation, specifically how residents prioritise the protection of tangible assets and livelihoods. Our findings indicate a clear preference for ‘self-sheltering’ in nearby reinforced private infrastructure, signalling a move towards localised self-reliance. To translate these insights into practice, we propose a shift from centralised public shelter models to integrated socio-technical individual resilient housing.

The following are the policy and implementation strategies:

- **Microgrant and technical assistance frameworks:** Governing bodies should pivot towards subsidising individual-level resilient housing. Actionable implementation involves standardising ‘resilience kits’ – blueprints for reinforced masonry, integrated sanitation and rooftop solar – linked to local microfinance institutions to ensure economic feasibility for low-income households.
- **Dual-purpose infrastructure policy:** Policy must mandate that all new local government buildings and schools be engineered as secondary refuge points, reducing the psychological barrier of leaving home for unfamiliar, crowded public shelters.
- **Cluster-based resilience planning:** Instead of isolated housing, planners should adopt ‘Integrated Resilient Settlements’. This involves zoning that co-locates housing with safe poultry and/or livestock pits and elevated granaries, directly addressing the ‘asset-loss’ anxiety that currently drives non-evacuation.

By integrating basic services with structural safety, DRR becomes a vehicle for socio-economic advancement rather than a temporary emergency measure. This approach shifts the burden from reactive evacuation to proactive, site-specific resilience.
